# The community resource management area mechanism: a strategy to manage African forest resources for REDD+

**DOI:** 10.1098/rstb.2012.0311

**Published:** 2013-09-05

**Authors:** Rebecca A. Asare, Andrew Kyei, John J. Mason

**Affiliations:** 1 Nature Conservation Research Centre, PO Box KN925, Accra, Ghana; 2 Forest Trends, 1203 19th Street, Northwest Washington, DC 20036, USA; 3 Wildlife Division, The Ghana Forestry Commission, PO Box MB434, Accra, Ghana

**Keywords:** Africa, community-based natural resource management, CREMA, Ghana, REDD+

## Abstract

Climate change poses a significant threat to Africa, and deforestation rates have increased in recent years. Mitigation initiatives such as REDD+ are widely considered as potentially efficient ways to generate emission reductions (or removals), conserve or sustainably manage forests, and bring benefits to communities, but effective implementation models are lacking. This paper presents the case of Ghana's Community Resource Management Area (CREMA) mechanism, an innovative natural resource governance and landscape-level planning tool that authorizes communities to manage their natural resources for economic and livelihood benefits. This paper argues that while the CREMA was originally developed to facilitate community-based wildlife management and habitat protection, it offers a promising community-based structure and process for managing African forest resources for REDD+. At a theoretical level, it conforms to the ecological, socio-cultural and economic factors that drive resource-users’ decision process and practices. And from a practical mitigation standpoint, the CREMA has the potential to help solve many of the key challenges for REDD+ in Africa, including definition of boundaries, smallholder aggregation, free prior and informed consent, ensuring permanence, preventing leakage, clarifying land tenure and carbon rights, as well as enabling equitable benefit-sharing arrangements. Ultimately, CREMA's potential as a forest management and climate change mitigation strategy that generates livelihood benefits for smallholder farmers and forest users will depend upon the willingness of African governments to support the mechanism and give it full legislative backing, and the motivation of communities to adopt the CREMA and integrate democratic decision-making and planning with their traditional values and natural resource management systems.

## 1. Introduction

Climate change poses a significant threat to Africa, and according to the Intergovernmental Panel on Climate Change (IPCC), reducing emissions from deforestation and forest degradation (REDD) not only offers one of the most cost-effective means of providing early mitigation, but also has the potential for poverty alleviation [1]. While forests and agriculture are addressed separately in international discussions in terms of projected risks, impacts, and adaptation and mitigation strategies, there is widespread recognition of the inter-connectivity of forests and agricultural system in Africa, and the integral role that people and society play in ecosystem patterns and processes [2].

Today, there are over a billion people living on the continent [3]. Africa has over 6.6 million km² of forest [4] and is reported to have 1.9 million km² of trees in agricultural lands [5]. Together, forest and agroforest landscapes make up more than a quarter of the continent. Depending on the country, 50–85% of the population is involved in agriculture, and depending on the context, agriculture is often cited as one of the major drivers of deforestation and degradation. Despite the fact that net annual forest loss has declined globally, Africa has had one of the world's highest rates of deforestation in recent years, with a net annual forest loss of 3.4 million hectares between 2000 and 2010 [6]. Thus, it is imperative that effective models and mechanisms are employed which can help us to reduce rates of REDD without compromising livelihoods or agricultural productivity. Given the scale of smallholder agriculture, these mechanisms will also need to be effective at building networks and platforms for aggregating large numbers of smallholder farmers and forest users, and at fostering long-term engagement that results in real climate mitigation benefits in addition to adaptive capacity.

To do this successfully requires generating a thorough understanding of the targeted forest landscapes and the key relationships that are at play between the biophysical resources (forests, trees and crops) and the associated social systems. Failure to understand or acknowledge these roles and relationships represents one of the greatest risks to successful management of forests and agroforests as part of a climate change mitigation strategy.

For example, policy development and implementation across the continent has had a very mixed track record within forestry and agriculture sectors, particularly in relation to the management of forest and tree resources, and community-based conservation efforts [7–9]. Often, communities and smallholders are marginalized, misunderstood or under-valued, compared with other actors and stakeholders in these processes; despite the fact that they tend to be the de facto resource users, managers and decision-makers. Thus, in an era when concerns over climate change are high and deforestation rates in Africa are alarming, the question of who can most effectively manage forest resources for successful outcomes, which provide economic, social and ecological functions and benefits at multiple scales, is paramount.

The literature has demonstrated that in Africa, communities and rural forest users can successfully manage natural resources, including forest and agroforest resources for multiple services and benefits [10–14] but outcomes largely depend on the context and the array of social factors and variables at work in these resource systems [15,16]. Where there is consensus that smallholder agriculture and forest-based livelihoods are among the main drivers of deforestation or degradation, it is imperative to understand what is driving smallholders' decision-making. From this standpoint, it is possible to consider how to effectively influence rural resource-use decision-making to support multiple ecosystem services and benefits, including REDD+.

### (a) REDD+ in Africa

For the 18 African countries with significant forest resources that are participating in either the UN-REDD Programme or the Forest Carbon Partnership Facility (FCPF) of The World Bank, reducing emissions from REDD (plus sustainable forest management, enhancement of carbon stocks and conservation) (REDD+), is a leading mitigation initiative that has the promise of providing benefits from emission reductions or removals. Since the launch of this international initiative, some researchers have argued that in Africa, REDD+ will be neither quick nor easy [17] and concerns have been raised over risks for local communities [18]. More broadly, much attention has been given to issues of social impacts on communities and rural resource users, and doubts have been raised about whether REDD+ can truly furnish transparency and equity while developing emissions reductions. It is widely recognized that in Africa, some of the most significant challenges to implementing REDD+ (as well as other mitigation activities such as climate smart agriculture or nationally appropriate mitigation actions (NAMAs)) may include issues surrounding tree tenure, land tenure and user rights [19]; good governance [20]; benefits and benefit-sharing arrangements [21,22]; aggregating smallholders across dynamic forest and agricultural landscapes [23] and technical capacity [19,20].

While the discourse surrounding REDD+ has been vibrant and frequently critical in nature, there has been a dearth of constructive, experienced-based ideas about how REDD+ can work in Africa, in light of the perceived challenges. Furthermore, few models have been presented which articulate bio-social approaches that have the potential to enable successful testing and piloting of potential REDD+ pathways. This paper, aims to help fill this gap by presenting the Community Resource Management Area (CREMA) mechanism and arguing that it offers a promising community-based structure and process for managing African forest resources for climate change mitigation and livelihood benefits.

Initially, the paper introduces the CREMA concept, describing the basic structure and process of developing a CREMA. It then presents the theoretical and practical elements of success and weakness, as well as lessons learned from practical experiences working with CREMA stakeholders. The paper then explains how the CREMA model evolved from a wildlife management tool to a mechanism for REDD+. Finally, the paper outlines how the CREMA mechanism helps one to solve some of the most complex challenges and risks associated with implementing REDD+ in Africa.

## 2. Ghana's community resource management area mechanism

The CREMA mechanism is an innovative natural resource management and landscape-level planning tool for community initiatives. It was developed by Ghana's Wildlife Division, an arm of the Forestry Commission, together with its partners, to support community resource management in off-reserve (un-gazetted) lands. CREMAs fill a critical gap by giving communities the right to manage and benefit economically from their natural resources. While Ghana's Constitution vests ownership of the land in the Stool or Skin (the traditional or customary leadership structures that preside over a particular ethnic group, clan or tribe and the associated land and resources) it gives the Government the right to manage the naturally occurring resources for economic gain [24]. This has resulted in a series of perverse incentives [25] that, over the decades, have tended to drive ‘illegal’ resource use and degradation or deforestation of the forest resources. The CREMA represents a profound policy shift by permitting communities, land owners and land users an opportunity to govern and manage forest and wildlife resources within the boundaries of the CREMA, and to benefit financially or in kind.

In Ghana, the CREMA process has followed a nearly 20 year evolution from an intellectual concept to an approved pilot initiative and finally to an authorized mechanism, which is now seeking full legal backing from Parliament. As originally conceived, the CREMA approach provided a mechanism by which the Wildlife Division could transfer authority and responsibilities for wildlife to rural communities. It denoted a geographically defined area endowed with sufficient resources where the people had organized themselves for the purpose of sustainable management of their natural resources. The aim was to encourage local people to integrate wildlife management into their farming and land management systems as a legitimate land-use option. The CREMA concept officially emerged from the 1994 Forest and Wildlife Policy, but it took the better part of a decade for communities to put it into action.

### (a) The CREMA structure and process

CREMA development is not a rapid process; typically taking at least 3–5 years until inauguration. Successful community-based management is an adaptive process [26] that requires patience and a sustained commitment from all stakeholders as community consensus-building and decision-making do not happen overnight and can be fraught with complexities. One of the greatest strengths, however, of the mechanism is that it is founded upon traditional or local beliefs and value systems, while being couched within a democratic decision-making and governance process. For example, many CREMA boundaries are drawn according to traditional area boundaries, and CREMA by-laws often incorporate or derive from local norms or traditional systems of forest and wildlife management.

All functional CREMAs come under a two-tiered governance structure, an approved constitution and rules and regulations, backing in the form of local government by-laws, the power to engage their own staff and the authority to generate revenue from natural resource management. In addition, CREMAs must have defined boundaries that are agreed upon by all stakeholder communities and the traditional leadership, upon which a long-term vision, goals, management plans, activities and regulations are agreed. As such, CREMAs represent a strong community structure that facilitates landscape planning, democratic decision-making, community-based governance and local design of benefit-sharing agreements for all stakeholders. A CREMA is officially inaugurated when the Ministry is sufficiently satisfied to issue an official Certificate of Devolution of rights over natural resource management to the local CREMA institution.

The CREMA development process usually begins with an initial assessment and consultation period in which an external stakeholder (NGO) or a government agency (Wildlife Division) works with community leaders to assess whether the site is a potential CREMA or not. Critical determinants include the community structure and level of organization, land tenure regimes in the target area, existing land-use practices and current uses of natural resources by the community(s) that may form part of the CREMA. If the results bode well for CREMA development, then the community leaders and traditional authorities must agree to engage in the CREMA process.

This is typically followed by a number of detailed studies including a socio-economic and ethnographic survey, a biological survey, an ethno-biological survey and an assessment (including mapping) of land uses, habitats and natural resource management systems. Widespread sensitization follows, culminating in the initiation of the process to build the CREMA.

The first step is to develop the CREMA management structure. Initially, this involves the creation of a community resource management committee (CRMC) in each CREMA community or in a cluster of communities. Committees typically consist of 5–13 men and women who are nominated or elected during a village-wide meeting, and who adequately represent the various sub-groups within the village. The role of the CRMC is to help envision the goals and objectives of the CREMA, to implement activities and to serve as the main liaison between the CREMA Executive Committee (CEC) and the individual community. Eventually, CRMC representatives and traditional leaders come together to draft a constitution. A constitution in the CREMA context is a social contract that sets out the organizational structure, defines the ‘community’ and its purpose and sets the basic rules and regulations that all will abide by. Following consultations with all of the communities that make up the CREMA, and with the Wildlife Division (Forestry Commission), the constitution is vetted and ratified at a final meeting with CRMC representatives and traditional leaders. Representatives from the community committees are subsequently elected to serve on the CEC, in addition to other co-opted resource persons. The CEC is the over-arching management body that directs and oversees CREMA operations and decision-making.

The next step is to define the CREMA boundary so as to determine the area within which the constitution is enforceable. This boundary, which defines the ‘community’, should be clearly marked as it will ultimately be backed by District Assembly by-laws. During this time, the CEC and the CRMC also engage in land-use planning, develop a strategy and set of activities (management plan) for the CREMA, and define the appropriate benefit-sharing arrangement for revenue that will be generated. These deliberations eventually culminate in the enactment of more detailed CREMA rules and regulations.

All CREMA stakeholders must agree upon a benefit-sharing arrangement that reflects their values, expectations and needs. Benefits usually include financial as well as non-financial resources, including payments at the individual or household level, access to information or agronomic resources, community development projects and scholarship funds. When CREMAs begin to generate revenue, transparent financial management is crucial. Multiple signatories on a local bank account, frequent oversight and auditing of accounts by the CEC and a third-party entity, and investment in trust funds, managed by a third party, are just some of the ways in which existing CREMAs have worked to foster financial transparency and accountability.

The final step before official recognition (inauguration) is for the CEC, traditional authorities, the Wildlife Division and the District Assembly to review all of the CREMA rules and regulations in the context of other national laws and District Assembly by-laws. The CREMA rules are then drafted as district by-laws and eventually presented for debate and ratification before the General Assembly of the District Assembly.

The final step is the inauguration of the CREMA and the issuance of a certificate of devolution by the presiding Minister, who gives the CEC the authority to manage its natural resources. This is not, however, the end of the process, but rather a shift from development to daily operations.

### (b) Theoretical and practical elements of success and weakness

At a theoretical level, the CREMA mechanism's applicability to community-based governance and management of forest resources stems from the fact that it conforms to a resource users’ multi-faceted decision-process. According to Firey [27], there is a biophysical, ethnographic and economic aspect to every resource-use decision. The physical environment influences how people use resources, and for a resource system to be sustainable, any practice within the system must be ecologically possible. Culture also bears a strong role in determining how people use resources, and any practice or resource system must be valued by a community or group in order to be adopted. Finally, each resource practice is influenced by the potential gainfulness or efficiency of that practice within the context of the entire resource-use system. Firey [27], therefore, argues that an optimal practice would be one which is ecologically *possible*, socio-culturally *adoptable* and economically *gainful*.

The CREMA concept directly reflects the principles of Firey's bio-social theory. It gives communities formal access and user-rights to the forest resources (ecologically possible), it is built upon traditional values and cultural systems (culturally adoptable), and it aims at generating financial and non-financial resources for communities and individuals within the CREMA (economically gainful). Depending on the scenario, the CREMA either supports and expands the scope of land-use practices and management decisions that already contain elements of sustainability (wildlife conservation through taboos and traditional hunting norms), or in an unsustainable situation (deforestation) it can entirely change the biophysical, economic and social conditions and resources that affect decision-making at the individual, community and social landscape level, opening up the possibility for different resource-use decisions and more sustainable outcomes.

Practical experiences, as elucidated in formal CREMA assessments [26,28], reviews [29] and the authors’ experiences also demonstrate that the combined ecological, ethnographic and economic elements of CREMAs are crucial to fostering successful outcomes. Successful CREMAs, that is, CREMAs which have either partially attained their goals and are on track to inauguration (and have not been abandoned) or received certificates of devolution tend to share the following ecological, socio-cultural and economic elements [26,28,29].

— Biophysically possible
  (i) The CREMA reduces threats to biodiversity and the environment, and/or biodiversity and the ecosystem remain stable.
— Socio-culturally adoptable
  (i) The CREMA is driven and demanded by the local people and communities.
  (ii) The CREMA is well integrated with traditional values and traditional systems.
  (iii) The CREMA builds real social capital and has strong and unwavering leadership that does not entertain nepotism and impunity.
  (iv) The CREMA is founded upon a constitution and associated by-laws, which are written and accepted by the people.
  (v) The CREMA receives backing at the local and national government levels that empowers management, enforcement of rules and regulations, and generation of revenue.
— Economically gainful
  (i) The CREMA has technically sound and consistent support during its development stage.
  (ii) The CREMA is economically self-sufficient and has a sustainable source of revenue; preferably multiple revenue streams.
  (iii) The benefit-sharing arrangement is defined by the communities themselves and tangible benefits are shared.

When the following elements are lacking (or risks are present), evaluations have shown that the CREMA can be weak or ineffective, and the chance that the CREMA will achieve or maintain its natural resource management and livelihood goals diminishes [26,28,29]:

— The community is nothing more than a passenger to a CREMA process, which is being defined and driven by an outside entity such as the government or an NGO (that may be constrained by funding and project time-frames).
— The institutions supporting a CREMA's development lack resources and capacity. A review of six CREMAs in Ghana's Western Region identified implementation challenges, including a lack of resources (human, financial and equipment) to support full CREMA development, poor communication and collaboration between outside institutions working to support CREMA development, and insufficient capacity to provide the necessary technical backstopping [28].
— The CREMA development process is rushed such that adequate time is not taken to determine the most appropriate decision-making infrastructure and other social tensions are not resolved.
— Lack of effective leadership and an absence of transparent, democratic decision-making and accountability.
— Strong beliefs in supernatural powers that affect people's perception of the threats to environmental and biological resources.
— Revenue or benefits fail to materialize, making up-front economic analysis of the proposed activities and the associated market opportunity essential.
— A substantial change in critical socio-cultural (new Chief, land tenure disputes) economic (demand for natural resources increases) or ecological variables (climate change) due to external or internal forces.

### (c) From wildlife to REDD+

In Ghana, early CREMAs were all focused on wildlife management and habitat protection, typically in the vicinity of protected areas. One of the most touristed CREMAs in Ghana—the Wechiau Community Hippo Sanctuary in Ghana's Upper West Region—has seen over 15 years of adaptive community-based management, resulting in successful management and revenue generation [26]. Early CREMAs developed revenues from eco-tourism and more recently from sustainable harvesting of non-timber forest products (NTFPs), with some CREMAs earning premiums for organic and conservation status NTFPs.

As of 2010, Ghana's Wildlife Division was officially monitoring 26 CREMAs, as listed in table 1. At that time, eight were in the process of being created, while 18 had been inaugurated and management rights devolved by the Ministry of Lands and Natural Resources of the CREMAs in table 1, the average CREMA covers 12 431.5 ha, ranging from 2046 to 40 000 ha and encompasses multiple communities or settlements.

**Table 1.** Description and status of active CREMAs being monitored by the Wildlife Division as of 2010. I, inaugurated; BC, being created.

name	district	area (ha)	no. of communities within CREMA	I/BC
1. Ayensu-Mmofrafadwen (Naptoman)	Wasa Amenfi West	9286	7	I
2. Asomase-Dadwen	Ellembelle	9550	4	I
3. New Adiembra	Ellembelle	3922	4	I
4. Ayewora-Anyinase	Ellembelle	4698	8	I
5. Sendu-Ansongkrom	Jomoro	5698	4	I
6. Ohiamadwen-Fiasoro	Jomoro	3707	4	I
7. Tweakor	Jomoro	9550	5	I
8. Amokwawsuazo	Jomoro	4520	9	I
9. Cocotown-Ghana Nungua	Jomoro	2828	4	I
10. Cape Three Points-Princess Town	Ahanta West	6353	11	I
11. Elluokrom	Bia	7743	11	I
12. Krokosua Hills	Juabeso	4580	10	I
13. Kwamebikrom Stool Lands	Bia	7277	10	I
14. River Asuopri	Bia	6133	4	I
15. Sureso-Pebase-Akyekyere	Wasa Amenfi West	9100	20	I
16. Murugu-Mognori	West Gonja	22 377	2	I
17. Kunlog (Jilinkon)	Sawla-Tuna-Kalaba	15 084	1 (plus satellite villages)	I
18. Yazori-Kaden	West Gonja	40 000	2	BC
19. Zukpiri Wildlife Sanctuary	Nadowli	4000	14	BC
20. Wechiau Hippo Sanctuary	Wa West	24 000	17	BC
21. Bodaa	Jaman South	2046	3	BC
22. Akyem-Pusupu	Nkwanta South	8768	3	BC
23. Boabeng-Fiema Monkey Sanctuary	Nkoranza North	7000	9	BC
24. Avu Lagoon Conservation Area	Keta, South Tongu, Akasi	30 000	15	BC
25. Asumura CREMA	Asunafo North, Asunafo South	35 000	19	BC
26. Afram Arm Manatee CREMA	Kwahu North	40 000	21	BC

Since Ghana began to engage in the REDD readiness process in 2007 through the FCPF, the question has been raised whether CREMAs could develop future carbon revenues. The Forestry Commission ultimately endorsed the principle of using the CREMA mechanism for managing carbon landscape projects, as evidenced by Ghana's REDD readiness preparation proposal (R-PP), which explicitly cites the CREMA mechanism as a means for implementing REDD+ demonstration projects and pilots [30]. No CREMA has realized emission reductions revenue yet, but a number of CREMAS are now exploring this possibility.

**Figure 1.**
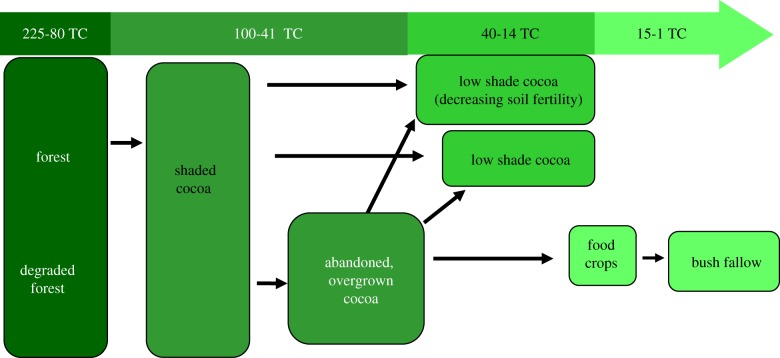
Land-use change pattern over past 30+ years in the Asumura CREMA and adjacent forest reserve landscape and estimated above-ground carbon stock ranges (TC/ha). (Online version in colour.)

**Figure 2.**
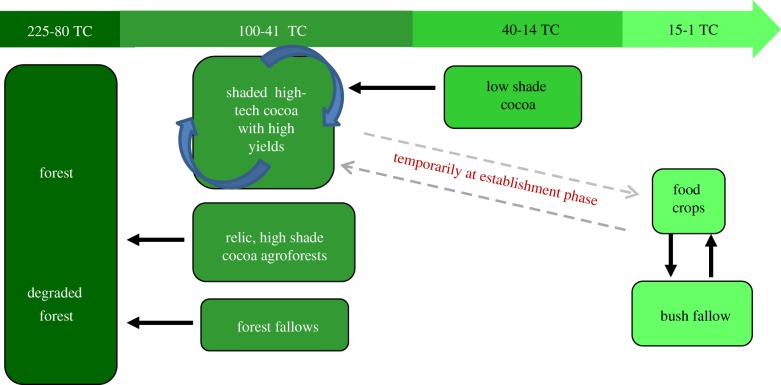
Desired climate smart cocoa land-use pattern and estimated above-ground carbon stock ranges (TC/ha). (Online version in colour.)

One such CREMA is the Asumura CREMA, which spans approximately 35 000 ha in the Asunafo North and Asunafo South Districts of the Brong-Ahafo Region. It constitutes 19 communities in a cocoa-growing landscape that lies adjacent to a chain of forest reserves—Subim, Ayum and Bonsam Bepo. Across Ghana's high forest zone, agricultural expansion and cocoa farming, in particular, have served as major drivers of forest degradation and deforestation [24]. In the light of these trends, the Asumura CREMA stakeholders are exploring whether carbon benefits can be generated by changing two basic baseline scenarios: (i) reducing cocoa farm expansion into the three forest reserves and (ii) reducing the rate at which low productivity cocoa farms with medium-to-high levels of shade are being converted to low shade, low productivity cocoa farms. Figure 1 depicts the current land-use change pattern (business as usual scenario) within the CREMA landscape and associated carbon stock estimates, whereas figure 2 depicts the ‘desired state’ for the Asumura CREMA landscape following CREMA development and project implementation, including the widespread adoption of climate smart, ‘high tech’ cocoa-farming practices that can double or triple cocoa yields [31]. The carbon stocks depicted in figures 1 and 2 were estimated using the Biomass Map of Ghana [32], coupled with non-random (intentional) biomass sampling in two dominant land-use types—cocoa farms (under three different shade regimes) and forest fallows. Specifically, twenty-four 1 ha nested plots were established to measure biomass and gather information about the previous land-use type.

Thus, the CREMA concept has evolved in Ghana from a mechanism that was specifically focused on wildlife management and habitat protection to one that is being adapted to support REDD+ project implementation and enable communities to manage forest and tree resources, in addition to wildlife, in the off-reserve landscape for climate mitigation and livelihood objectives.

## 3. CREMA as a strategy to manage African forest resources for REDD+

Climate change poses a significant threat to Africa and deforestation rates have increased in recent years. Mitigation initiatives such as REDD+ are widely seen as potentially efficient ways to generate emission reductions (or removals), conserve or sustainably manage forests, and bring benefits to communities, but REDD+ faces many challenges in Africa, and effective implementation models are lacking. As of 2011, there were very few smallholder/community-based REDD+ experiences to learn from, and yet opportunities for learning are crucial in light of the complexities to implementing REDD+, when compared with other types of carbon projects or to REDD+ in other parts of the world. Across the continent, the majority of forest carbon projects are for afforestation/reforestation [33,34]. Peters-Stanley [33] explains this discrepancy by the fact that the context of REDD projects is significantly more challenging than that of other types of carbon projects, and progress is slow in Africa especially because, ‘REDD projects are intertwined with some of the world's knottiest issues’, including unclear or overlapping land tenure, shifting subsistence agriculture, population and economic growth pressures, legal and illegal extraction of forest resources, lack of enforcement agency coordination and resources, and the absence of land management plans [33].

Although not originally developed for REDD+, lessons from the CREMA experience are highly relevant for REDD+ projects aimed at furnishing benefits to smallholders and communities. The CREMA process is also compatible with the process of developing a REDD+ project, and the mechanism itself has the potential to provide a neat solution to a number of the challenges to implementing REDD+ (as well as to other types of carbon projects), especially in=countries where complex land and tree tenure regimes prevail.

### (a) CREMA synergies and solutions to REDD+ implementation challenges

The CREMA development process and the mechanism itself help one to solve some of the main social, tenure, technical and benefit-sharing challenges associated with implementing REDD+ in community-based carbon projects in Africa. As ensuing points show, CREMAs have the potential to play a consistent role in negotiations with traditional leaders, farmers and communities, and in implementing the project activities. They can help one to ensure compliance through the development of by-laws and monitoring activities, and they are attuned to structuring benefit-sharing systems. The process and structure of the CREMA combine to enable democratic decision-making and problem solving, resulting in strong social cohesion that significantly increases the likelihood of permanence and reduces potential leakage. From an investor's standpoint, this reduces the overall internal risk of the project. Although carbon rights are still to be fully clarified, it is expected that the government will vest CREMAs in Ghana with full or partial carbon rights (or the right to benefit from the emissions reductions or enhancements), eliminating one of the strongest impediments to carbon projects in Africa. The following points describe these advantages, solutions and synergies in greater detail.

#### (i) Clear project boundaries

Setting project boundaries that conform to the boundaries of a CREMA (or to multiple CREMAs) is an efficient and socially relevant means of determining REDD+ project boundaries. It simplifies justification of project boundaries and gives local backing to the boundary demarcation decision. It also ensures clear decision-making within the boundaries, eliminating potential overlap of traditional jurisdictions or fragmentation of social systems and landscapes.

#### (ii) Smallholder aggregation

In many places in Africa, forest and agroforest landscapes are inhabited and used by hundreds if not thousands of smallholder farmers and forest users. Thus, a successful carbon project and/or community-based forest management project will necessarily have to bring together many stakeholders, of a potentially heterogeneous nature, under a common management agenda. The CREMA establishment process and structure inherently provide a means by which to aggregate and build consensus at multiple social scales across a landscape. The participatory and adaptive nature of the CREMA creates opportunities to address differences of opinion, and to support democratic decision-making processes that are backed-up by traditional values and by-laws. The result is that the CREMA inherently brings together large numbers of resource users in an efficient manner, without becoming bogged down by the need to confer with each individual or household in the project area. By virtue of the governance structure, the CREMA leadership has the authority to finalize and implement decisions on behalf of all members (individuals, communities and traditional leaders).

#### (iii) Free, prior informed consent

The CREMA constitution is developed through an extensive participatory process that involves all communities and relevant stakeholders. This process and subsequent ratification of the constitution goes a long way in ensuring free, prior informed consent (FPIC), a requirement of REDD+ and other types of carbon projects.

#### (iv) Permanence and leakage

The CREMA operates through a locally approved institutional structure that informs and oversees the day-to-day governance of the CREMA. This structure should result in strong social cohesion and problem-solving capacity, which supports the permanence of the carbon asset, and reduces potential leakage. Furthermore, CREMA land-use planning leads to the articulation of rules, as well as monitoring and enforcement plans that should support permanence and discourage leakage. CREMA rules and regulations are given legislative backing through local government by-laws, which permits actual enforcement. When problems of permanence or leakage arise, community and CREMA leaders can rely upon the power of collective social pressure or enforcement of the by-laws to address the problem.

#### (v) Letter of no objection from REDD focal point

CREMAs must follow a process of formal review, approval and oversight by the national government; in Ghana's case, through the Wildlife Division of the Forestry Commission. In Ghana, the REDD+ Secretariat is housed at the Forestry Commission and has issued its support for using CREMAs to implement REDD [24]. Thus, the likelihood of receiving a letter of no objection from the REDD Focal Point is quite high as the government has been a high level participant in the process.

#### (vi) Land tenure, tree tenure and rights to biomass

In much of Africa, forest resources are managed in a pluralistic framework in which both statutory and customary laws prevail, either formally or informally [35]. In Ghana and other countries, title deeds are relatively rare and land disputes can be common [36], greatly increasing the internal risk assessment for carbon projects. The lack of clarity with regard to land tenure, tree tenure, forest/tree management rights and ownership of biomass can be one of the main stumbling blocks for forest carbon projects in Africa. The CREMA provides a unique loophole to help solve these tenure and management barriers; at least until governments are ready to directly tackle these issues with respect to carbon mitigation. For example, the certificate of devolution of Authority from the government gives CREMA authority the right to manage the forest resources, including biomass, within the CREMA boundaries.

#### (vii) CREMA as a legal entity

While CREMAs are supported by Ghana's Forest and Wildlife Policy and Ministerial consent can be given to individual CREMAs, the CREMA mechanism has yet to receive explicit legislative authority from Parliament. This is an important final step in conferring CREMAs with full rights to manage and benefit from the forest resources, and remains a weakness of the mechanism. Until such time as the Parliament approves the new Forest and Wildlife Bill, which includes language on CREMAs, CREMAs have the option, under Ghanaian law, to incorporate as a legal entity (corporate) that is permitted to enter into contracts on behalf of its membership, and can serve as an effective structure for the conferment of rights and benefits [37].

#### (viii) Potential revenue stream

REDD+ or other carbon finance initiatives offer a potentially new source of revenue for CREMAs. Financial and non-financial benefits from carbon projects are highly compatible with the CREMA's more traditional sources of revenue and non-financial benefits, including harvesting of NTFPs, eco-tourism and wildlife management.

#### (ix) Benefit sharing

CREMA communities and authorities delineate their own benefit-sharing arrangements that are responsive to the CREMA stakeholders’ values, perceptions of equity and needs. Thus, benefit-sharing arrangements are internally defined as opposed to being externally imposed. It must be noted, however, that national benefit-sharing legislation or tax laws may have implications for the CREMA's benefit-sharing formula.

## 4. Recommendations

Proponents and stakeholders of REDD+ projects and programmes have much to gain from exploring and ultimately adopting CREMA (or CREMA-like mechanisms) as a strategy to enable REDD+ implementation on the ground. There are myriad synergies between REDD+ and the CREMA mechanism. As this paper has tried to demonstrate, implementation of REDD+ in Africa is confronted by some critical implementation challenges and risks (including definition of boundaries, small-holder aggregation, permanence and leakage, land tenure and rights to biomass, achieving FPIC and benefit-sharing), to which the CREMA mechanism and process can offer some neat solutions and risk reduction. Equally, the lack of sustainable and real economic revenue streams for community-based natural resource management and/or conservation projects has been a perpetual weakness, and in this respect, REDD+ and other climate mitigation strategies can bring very interesting performance-based benefits (both in cash and kind) to the table to dramatically change this scenario.

The authors do not purport that implementing a CREMA is necessarily easy; just like with REDD+ projects, constituting a CREMA can be complex and time-consuming. But the economic and ethnographic overlap between CREMAs and REDD+ suggests that in tandem, the likelihood of realizing emissions reductions or removals (as well as biodiversity conservation and other ecosystem services) and consequently producing real livelihood benefits is much higher. Given the alarming rate of deforestation in Africa, the fact that smallholder agriculture is one of the major drivers of deforestation, and the urgency with which climate mitigation actions are required, community-based forest governance and management models are needed that can reduce rates of REDD (and furnish carbon stock enhancement) without compromising livelihoods or agricultural productivity. This paper recommends the CREMA mechanism as one such tool.

In reality, however, the ability to use the CREMA as a forest management and REDD+ strategy in Ghana and beyond will depend upon a number of key factors, including: the responsiveness of African governments to this opportunity; policy makers’ willingness to adopt and adapt the strategy to the national and local context; the government's ability to provide legislative backing to such a mechanism; communities’ willingness to participate and to integrate a democratic decision-making structure and land-use planning process with their traditional values and natural resource management systems; and NGOs or other institutions’ willingness to provide a long-term supporting role. These are neither simple nor easy responsibilities, but the potential social, ecological, climate and economic benefits appear to far outweigh these challenges, and could ultimately take African countries a step closer to finding a long-term strategy to manage forest and tree resources for REDD+ and livelihood development.
